# Variation of tension in the long head of the biceps tendon as a function of limb position with simulated biceps contraction

**DOI:** 10.4103/0973-6042.68411

**Published:** 2010

**Authors:** Gregory G. Gramstad, Benjamin W. Sears, Guido Marra

**Affiliations:** Rebound Orthopaedics, 6702 SW Canyon Crest Dr, Portland, OR 97225, USA; 1 Department of Orthopaedic Surgery and Rehabilitation, Loyola University Stritch School of Medicine, 2160 S. 1^st^ Ave., Maywood, IL 60153, USA

**Keywords:** Cadvaric study, glenohumeral joint, limb position, long head biceps tendon, SLAP lesion

## Abstract

**Purpose:**

This study was designed to quantify tensile forces within the intra-articular long head of the bicep tendon (LHBT) under conditions of passive limb positioning and physiologic load, which simulate contraction of the LHBT.

**Materials and Methods:**

A force probe was inserted into the intra-articular LHBT, just distal to its supra-glenoid origin, in six fresh-frozen cadaveric specimens. Initially, specimens were manually manipulated through 30 glenohumeral joint positions, combining humeral rotation and elbow/forearm position. In the second phase, a 55 N tensile load was applied through the LHBT in 18 limb positions. Intra-tendinous tension was recorded in all positions under both conditions.

**Results:**

External humeral rotation significantly increased tension with glenohumeral forward flexion (*P*<0.0001). Conversely, internal humeral rotation significantly increased tension with glenohumeral abduction and extension (*P*<0.0001). A position of glenohumeral extension and internal rotation, with the elbow extended and forearm pronated, produced the highest tension in the intra-articular LHBT (*P*<0.0001). Under applied load conditions, observed LHTB tension was not statistically different in any glenohumeral position (*P*=0.1468, power = 88%). The greater tuberosity was noted to impinge on the force probe in forward flexion and internal rotation in two specimens.

**Conclusions:**

Variable tensile forces are seen in the intra-articular LHBT as a function of both limb position and simulated biceps contraction. Our findings provide a thorough data set that may be used to help substantiate or refute current or future hypotheses regarding LHBT function, pathology, and clinical tests.

**Clinical Relevance:**

Identifying positions of glenohumeral motion, which affect LHBT tension will provide an anatomic basis for clinical tests proposed to be for diagnosing LHBT lesions, including superior labral anterior and posterior tears.

## INTRODUCTION

The functional role of the long head of the bicep tendon (LHBT) in glenohumeral motion and stability has not been clearly established.[1–7] Some studies have suggested that the LHBT may function as a weak humeral head depressor in rotator cuff deficient shoulders, whereas others suggest that it may contribute to dynamic shoulder stability, particularly in overhead athletes.[4,6,8,9] It is likely that both the tension and position of the LHBT, relative to the humeral head and glenoid, affect its ability to function effectively as a humeral head depressor or stabilizer. A thorough understanding of LHBT tension, relative to position, may also provide both insight into the etiology of LHBT pathology and an anatomic basis for clinical tests designed to diagnose lesions of the LHBT.

Painful lesions of the LHBT can arise from both traumatic and degenerative etiologies and has significant clinical implications. The mechanisms leading to these lesions have remained a source of interest.[8,10–12] As well, many clinical tests have been described to diagnose lesions of the LHBT, including superior labral anterior and posterior (SLAP) tears, with varying degrees of specificity and sensitivity. The anatomic basis for these tests is often hypothesized, but has not been tested biomechanically. Several more recent studies have called to question the clinical accuracy of these tests, as reported by the original authors.[13–15]

The purpose of this study is to measure tension in the intra-articular LHBT as a function of limb position, both with and without an applied biceps load. We hypothesize that positions that lengthened the biceps muscle will increase tension in the LHBT and that a simulated contraction of the biceps muscle will further increase tension in those limb positions. We hope to provide a thorough data set that may be used to help substantiate or refute current or future hypotheses regarding LHBT function, pathology, and clinical tests.

## MATERIALS AND METHODS

Six fresh-frozen unilateral cadaveric forequarter upper extremities, ranging in age from 49 to 76 years, were used for testing. The specimens were dissected free of skin and deltoid. The biceps was grossly inspected and left intact. A static load of 22 N was applied to the tendons of the subscapularis, supraspinatus, and the infraspinatus/teres minor. This load has been used in previous studies to replicate rotator cuff tension and maintain glenohumeral concentricity during limb positioning.[16–18] The scapulae were potted in polymethylmethacrylate (PMMA) so that the plane of the glenoid was perpendicular to the floor and the scapular body was forward flexed 20°.

None of the specimens had evidence of advanced arthritic disease, unstable labral tears, or visible tears of the rotator cuff. There was no subluxation or tearing of the LHBT in any specimen. One specimen had hypertrophy of the LHBT in the bicipital groove, which did not cause restriction of motion through the bicipital groove. Two specimens had deficiency of the anterior superior labrum, and the bicep tendon was noted to arise from the supraglenoid tubercle and the posterior labrum equally.

**Figure 1:**
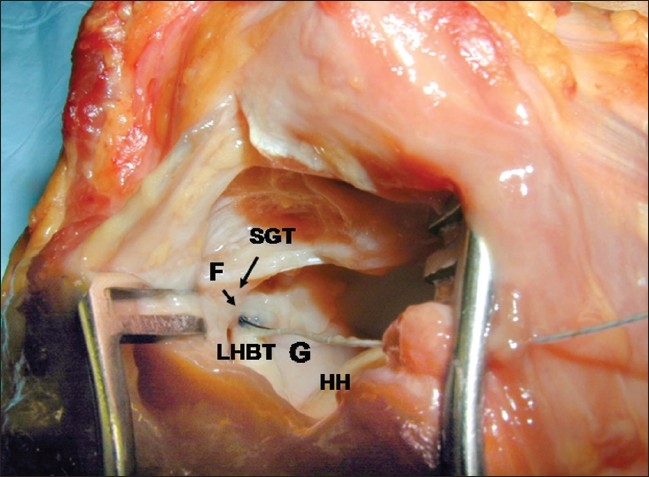
The IFP was inserted entirely into the substance of the LHBT, just distal to the confluence of the labral and tubercle origins of the tendon, and sutured in place. LHBT: Long head biceps tendon; F: Force probe; G: Glenoid; HH: Humeral head; SGT: Supraglenoid tubercle

Using loupe magnification, through a small rent in the rotator interval, an intra-tendinous force probe (IFP) (Microstrain, Burlington, VT) was inserted entirely into the substance of the LHBT, just distal to its labral origin [Figure 1]. Tension was measured in Newtons. The characteristics of this probe have been previously published, and its application in this study followed these guidelines.[19]

### Limb positioning

Poppen *et al*. described the glenohumeral-to-scapulothoracic ratio in shoulder motion which indicates that 90° of *in vivo* elevation is equivalent to 60° of glenohumeral elevation.[20] In this study, full elevation was defined as 60° of glenohumeral elevation in the scapular plane. *In vivo*, the chest wall prevents complete adduction of the glenohumeral joint. Therefore, we defined adduction as 20° of glenohumeral elevation. From 60° of elevation in the scapular plane, forward flexion was defined as further horizontal adduction of 60°. Coronal plane abduction and extension are defined as 30° and 60° of horizontal abduction, respectively, from elevation in the scapular plane. Three wire markers were placed in the acromion to guide horizontal glenohumeral motion from the plane of the scapula.

Humeral rotation was established using the bicipital groove[21] and the forearm as guides. A large threaded pin, placed into the humerus, provided the means to control rotation, whereas intramedullary instrumentation of the ulna and radius allowed for elbow flexion/extension and forearm supination/pronation to be controlled.

### Test protocol

The IFP was zeroed with the elbow in maximum flexion and supination. All manual limb motion began from a defined starting position of 60° of elevation and neutral humeral rotation. In this position, with the elbow in full flexion and the forearm supinated, it was observed that the biceps tendon was under no tension and was flaccid in the groove. Each specimen was taken through three testing runs, in which order of change in glenohumeral positions were randomized in the first and third runs, and the second run was conducted in reverse order. At each glenohumeral position (elevation, adduction, forward flexion, coronal plane abduction, and extension), six data points were recorded [Table 1]. The probe was re-zeroed between points, tension was recorded in three standard humeral rotations (neutral and maximal internal/external rotation for each specimen obtained without pressure), each with two positions of the forearm (maximum elbow flexion and forearm supination, or maximum elbow extension and forearm pronation) for every glenohumeral position. The limb was returned to the starting position before testing in another glenohumeral position. Recordings of tension were made in 30 limb positions.

**Table 1:** Summary of limb positions

Glenohumeral position	Humeral rotation	Forearm passive	No applied load	Applied load	Relevant positions
					
Forward flexion	Neutral	F/S	1	1	
		E/P	2		
	Internal	F/S	3	2	
		E/P	4		O’Brian active compression test
	External	F/S	5	3	
		E/P	6		
					
Adduction	Neutral	F/S	7	4	Yergason’s
					Supination sign
		E/P	8		
	Internal	F/S	9	5	
		E/P	10		
	External	E/S	11	6	
		E/P	12		
600 Elevation	Neutral	F/S	13	7	
(900 *in vivo*)		UP	14		
	Internal	F/S	15	8	
		E/P	16		
	External	F/S	17	9	
		E/P	18		
800 Elevation	Neutral			10	
(1200 *in vivo*)					
	Internal			11	
	External			12	Biceps
					load II test
Abduction	Neutral	F/S	19	13	
		E/P	20		
	Internal	F/S	21	14	
		E/P	22		
	External	F/S	23	15	Cocking phase of throwing
		E/P	24		
Extension	Neutral	F/S	25	16	
		E/P	26		
	Internal	F/S	27	17	
		E/P	28		
	External	F/S	29	18	
		E/P	30		

Thirty positions were tested in specimens without an applied biceps load (labeled 1-30), and 18 positions were tested in specimens with an applied biceps load (labeled 1-18). All elevation occurred in the scapular plane. “No applied load” refers to specimens tested passively without the addition of applied biceps load. “Applied load” refers to specimens tested with a 55 N load applied to the LHBT to simulate a biceps contraction. F: Flexion, E: Extension, P: Pronation, S: Supination

**Figure 2:**
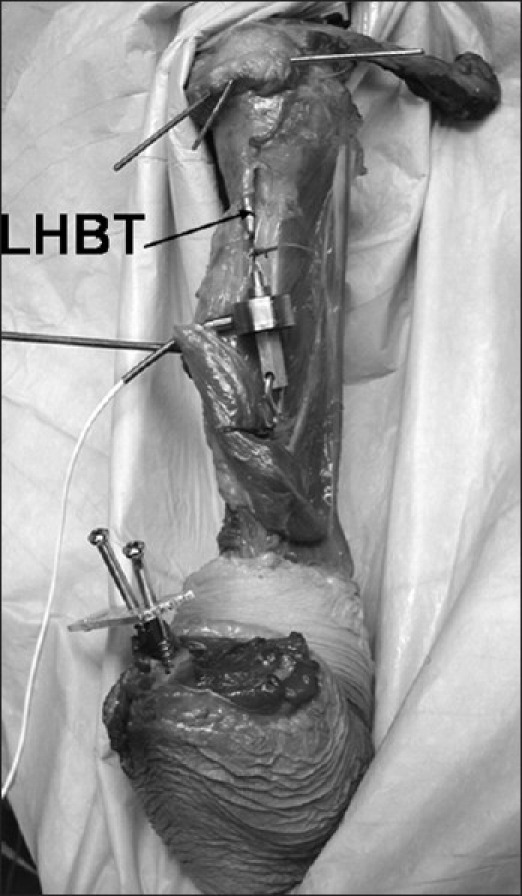
Set-up of applied load condition. Wire markers in the acromion guide horizontal positioning. With elbow extension, load is transmitted to the LHBT through the load cell

In the next phase, a 55-N load was applied to the LHBT to simulate a biceps contraction. This load has been calculated to be the force generated by the LHBT and has been used in previous published studies.[18,20,22] A load cell was sutured to both the proximal LHBT, distal to the intertubercular groove, and the distal bicep tendon with the elbow flexed [Figure 2]. The biceps was loaded to 55 N for 30 cycles prior to testing. After glenohumeral positioning and humeral rotation were set, passive elbow extension lengthened the biceps-load cell unit until the load cell reported 55 N, at which point tension within the LHBT was recorded. This apparatus allowed for the tensile force to be generated in line with the native bicep in all positions of glenohumeral motion and humeral rotation. Data points were collected in 19 positions [Table 1].

For both phases of the experiment, the order of the glenohumeral positions was randomized and three trials were run. The first and third trials were conducted in randomized order, and the second trial was run in reverse order.

After testing, the specimen was dissected to examine the glenohumeral joint, the capsule, the rotator cuff, the LHBT at the supraglenoid tubercle and in the groove, and the superior labrum. Anatomic variants and pathology were noted.

### Data analysis

Data analysis began by averaging the three trial runs at each position. The data were then analyzed using PROC GLM in SAS, version 8 (SAS Institute, Inc., Cary, NC). For the first analysis, under the passive motion condition, a randomized block design was employed. A factor of “position,” which is each of the individual manipulations of the arm, had 30 levels. Each specimen was a block, giving six blocks. An *F*-test of “position” was significant with *P*<0.0001. Contrast statements were used to do the comparisons of elbow flexed to extended positions. An overall alpha of 0.05 was adjusted for the 15 multiple comparison using Bonferroni. The alpha for each of the 15 comparisons was 0.05/15 = 0.00333. All subsequent comparisons were made using data from the elbow extended/pronated position.

At each glenohumeral position, a comparison of internal to external rotation was made using contrast statements. An overall alpha of 0.05 was adjusted for the five multiple comparisons giving an alpha of 0.01 for each individual comparison.

Several contrast statements were used to examine the five highest tension positions, one from each glenohumeral position. The position of the highest tension was compared to the average of the next four highest positions. A pairwise comparison was then used to confirm that the position of highest tension was significantly higher than the position of next highest tension.

The data from the applied load condition were also analyzed using PROC GLM, with the same randomized block design. An *F*-test of “position” on this data was not significant, so no additional testing on pairs of means was done.

A *post hoc* power analysis was performed using NCSS-PASS software (NCSS, Kaysville, UT). The analysis used means and standard error from the actual data and an ANOVA run for a sample size of six specimens.

The position of the IFP was visually verified to be consistent among all specimens. However, because the magnitude of absolute tension recorded for each specimen would be intrinsically affected by subtle differences in the position IFP placement within the tendon and by differences in tendon diameter, data were normalized as a percentage change from the starting (resting) position in order to use each specimen used as its own control.

## RESULTS

### Passive motion

**Figure 3:**
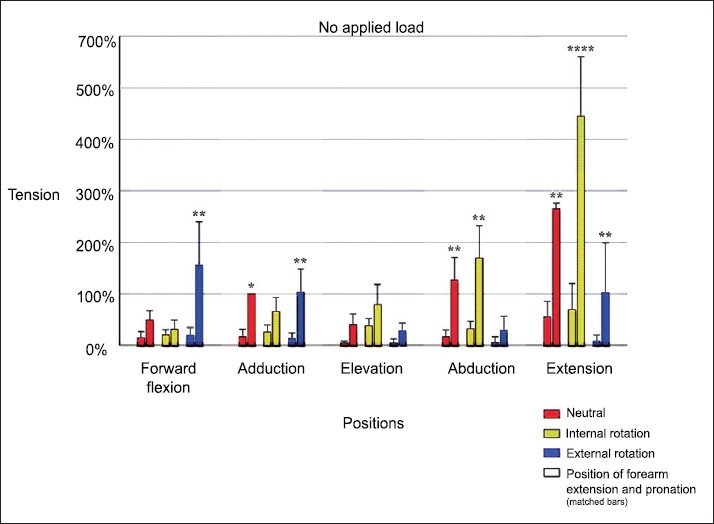
Long head biceps tension as a factor of limb position (no applied load). Resting position is defined as 100% tension. Significant increases in tension were noted in glenohumeral adduction with external rotation (*P*<0.0033), forward flexion with external rotation (*P*<0.0001), abduction with neutral (*P*=0.0007) and internal rotation (*P*<0.0001), and extension with neutral (*P*<0.0001), internal (*P*<0.0001), and external rotation (*P*=0.003). Elbow extension with forearm pronation increased tension in all glenohumeral positions compared to the flexed and supinated position (*P*<0.0033)

Figure 3 describes long head biceps tension as a factor of limb position (no applied load). With resting position defined as 100% tension, significant increases in tension were noted in glenohumeral adduction with external rotation (*P*<0.0033), forward flexion with external rotation (*P*<0.0001), abduction with neutral (*P*=0.0007) and internal rotation (*P*<0.0001), and extension with neutral (*P*<0.0001), internal (*P*<0.0001), and external rotation (*P*=0.003). Elbow extension with forearm pronation increased tension in all glenohumeral positions compared to the flexed and supinated position (*P*<0.0033). This increase was significant in seven of the 15 glenohumeral positions. When the elbow was flexed and the forearm supinated, the tension was never observed to be greater than the resting position, regardless of the glenohumeral position or humeral rotation.

The effect of humeral rotation was shown to be significant in forward flexion, coronal plane abduction, and extension (*P*<0.0001). In forward flexion, external rotation increased the tension over internal rotation (>500%) while the converse was true for abduction (>600%) and extension (>330%).

Glenohumeral extension with internal humeral rotation, combined with elbow extension and forearm pronation was the position that proved to have the highest tension over all other positions (*P*<0.0001). Average tension in this position, as recorded by the IFP, was 3.241 N (range 2.14-3.73 N). This limb position created more tension than abduction/internal rotation and forward flexion/external rotation, which were the next higher tensions, by a factor of two and a half.

### Applied load

**Figure 4:**
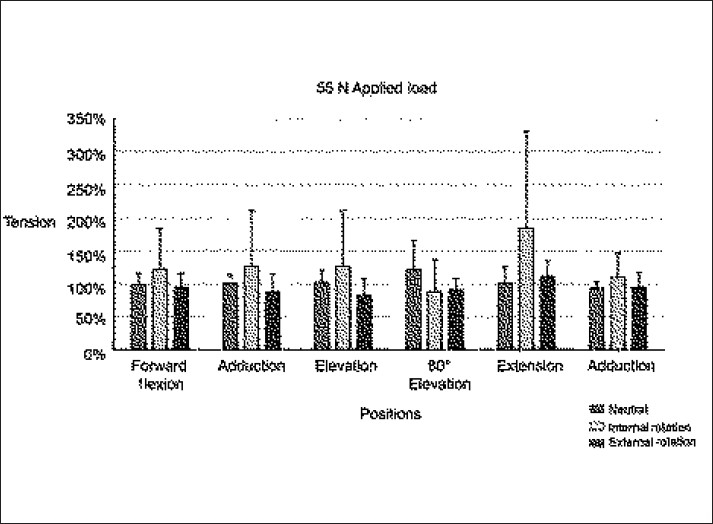
Long head biceps tension as a factor of limb position (with applied load). Resting position is defined as 100% tension. With application of a 55-N load, no position of the limb was found to significantly increase the intra-articular LHBT tension relative to the resting position (*P*=0.1468, power = 88%)

Figure 4 demonstrates long head biceps tension as a factor of limb position (with applied load). Resting position is again defined as 100% tension. When a 55-N load was applied, no position of the limb was found to significantly increase the intra-articular LHBT tension relative to the resting position (*P*=0.1468, power = 88%) [Figure 4]. Average tension in adduction and neutral rotation was 3.933 N (range 2.13-6.41 N).

### Greater tuberosity internal impingement

Uncharacteristically, high values were noted in two specimens under the applied load conditions in forward flexion with internal rotation. One specimen was producing load values approaching the yield point of the IFP (16 N). Normal values for other specimens were in the range of 3-6 N. The testing of this specimen was halted before completion, and a dissection was performed. With the limb in forward flexion, internal humeral rotation caused a mechanical compression of the probe between the greater tuberosity and the anterior-superior glenoid. The data from this seventh specimen were incomplete and were not included in data analysis. A second specimen was also noted to have uncharacteristically high values with internal rotation in forward flexion. However, since the values were not approaching the limits of the IFP, data collection was completed and reported. Dissection of this specimen post-testing revealed similar findings.

## DISCUSSION

The aim of this study was to investigate tensile forces experienced within the LHBT just distal to its supraglenoid origin as a function of limb position, both with and without a simulated biceps contraction. Our findings confirm the hypothesis that positions which lengthen the biceps muscle, also increase tension in the LHBT. Elbow extension and forearm pronation predictably increase tension over passive elbow flexion and forearm supination in all positions tested. This increase was statistically significant in positions resulting in the bicipital groove being rotated away from the superior glenoid. With the limb in forward flexion, external rotation of the humerus rotates the bicipital groove laterally, away from the glenoid. The LHBT must, therefore, course around the circumference of the humeral head, lengthening the biceps, and increasing tension. Conversely, internal humeral rotation in forward flexion aligns the bicipital groove with the superior glenoid and the tension is decreased. With the limb in coronal plane abduction or extension, internal rotation lengthens the biceps by rotating the bicipital groove anteriorly and inferiorly away from the superior glenoid, again causing the LHBT to course around a greater circumference of the humeral head resulting in increased tension.

Passive glenohumeral extension with internal rotation, combined with elbow extension and forearm pronation, resulted in the highest tension in the LHBT compared to all other limb positions. This position is termed the “reach back” position, as it correlates to the position of reaching into the back seat of a car or reaching back to place the limb into a sleeve.

In addition, our findings indicate that a simulated biceps contraction of 55 N results in a magnitude of tension greater than that produced by any passive limb positioning and that the tension produced by a simulated biceps contraction is not increased further by any positioning of the limb. This suggests that the effect of associated arm positioning on intra-articular LHBT tension is less than that of simulated full biceps muscle contraction. Therefore, it may be hypothesized that the efficacy of clinical tests that recreate pain by resisting a biceps contraction in a certain position of the limb may not be explained by tension alone, since tension would be the same in any position tested, according to our results. It is quite likely that the direction of tension and/or the impingement of a displaced lesion also play a significant role in the generation of symptoms.

Follow-up studies on the accuracy of clinical tests designed for diagnosing type II SLAP lesions have not reproduced the same high degree of sensitivity and specificity as the original reports.[13–15] It could be hypothesized that no single clinical test can be the “best test” for all type II SLAP lesions because of the variability in etiology and location of type II SLAP tears.

Since type II SLAP, lesions may have a more anterior or posterior detachment, some tests may be more efficacious in particular patient populations with certain types of SLAP lesions. Andrews’ resisted supination external rotation test was performed primarily in overhead athletes.[23] A positive test recreates symptoms with resisted supination as the arm is brought into the late cocking position of throwing. It was hypothesized that this test recreates the “peel-back” mechanism for posterior SLAP tear formation, as observed in throwers.[24] Our results do not refute this potential mechanism, as maximal biceps contraction was seen to cause maximal tension in the LHBT, regardless of limb position. While this test may be efficacious at diagnosing SLAP lesions in throwers, it may be less reliable at diagnosing SLAP variants with a more anterior detachment. It could be hypothesized that O‘Brien’s active compression test may be better designed to displace and compress lesions with a more unstable anterior detachment.

O‘Brien’s active compression test[25] is described with the arm in forward flexion, with an additional 15° of horizontal adduction and elbow extension. With the arm in full internal rotation, a downward force is resisted (positive position). The arm is then fully supinated, and the downward force is again resisted (negative position). A positive test will produce pain or a painful clicking inside the joint in the positive position, with relief in the latter. The author’s hypothesized a displacement-compression mechanism for the positive test.

A recent study evaluated active and passive tension in the LHBT in the two positions of the O’Brien active compression test and found tension to be higher in the negative position (external rotation and forearm supination), concluding that the clinical test was not validated anatomically.[26] However, in this study we observed a direct compressive mechanism of the anterior greater tuberosity on the force probe in forward flexion and internal rotation (positive position) in two out of six (33%) specimens. This observation may support the findings of the initial investigators who observed compression of the SLAP lesion in the positive position of the active compression test during arthroscopic evaluation.[27] The reason that impingement of the probe did not occur in all specimens may either be secondary to subtle differences in the location of force probe placement within the tendon, or because specimen positions did not include the 15° of horizontal adduction described in the original active compression test description. In similar fashion, in the clinical setting, the size and location of the SLAP lesion and its potential for displacement could affect the efficacy of the active compression test.

There are several limitations to this study. One limitation is the possible effects of tissue visoelastic properties that occur during repetitive stress testing of cadaveric soft tissues. We randomized the order of the tested positions for each specimen and performed the second trial in reverse. This was done in an attempt to decrease the effect that stress relaxation would have on our results if one position was always tested last. We evaluated the raw data of several specimens and noted that there was no detectable decline in tension by the third and final run. In the second phase of the study, the biceps was loaded to 55 N for 30 cycles before testing began to minimize the effects of tissue creep. Another potential limitation of this study is placement of the force probe. This study relied on the use of a simple longitudinal force transducer placed into the intra-articular LHBT, thereby specific local changes to tendon tension outside of this probe position, such as those occurring in the biciptial groove, may have been overlooked. Intra-articular tensile forces cannot be assumed to represent the forces experienced by the LHBT within the bicipital groove owing to the effects of osseous compression or torsional forces as the tendon enters the joint. This may potentially impact patient clinical presentation or influence clinical significance of pain associated with various positions of the shoulder.

However, while alternate forces along the LHBT certainly exist and could result in clinical symptoms, this purpose of this study was to examine forces at the LHBT origin because of the clinical implications and surgical associations made with this position of tendon. Finally, since the IFP measures the tension exerted upon its walls by the surrounding tendon fibers, subtle differences in placement position and varying diameters of the LHBT precluded a direct comparison of absolute tension between specimens. Instead, each specimen was used as its own control and tension was reported as a percent variation from a reference position. The resting position of adduction and neutral rotation with elbow extension and forearm pronation was chosen for the reasons previously mentioned. Several other reference positions were evaluated during calculation to ascertain the reliability of the resting position, and the data did not show significant variation from what we report.

## CONCLUSIONS

The results of this study indicate that variable tensile forces are seen in the intra-articular LHBT as a function of limb position and applied load (simulated biceps contraction). These forces have implications in forming hypotheses regarding clinical tests used to diagnose biceps-labral pathology. We found the “reach back” position consisting of glenohumeral extension with internal rotation, elbow extension and forearm pronation, significantly increased tension forces within the intra-articular LHBT more than any other passive limb position. The tensile forces generated by a simulated biceps contraction were not affected by limb position and were greater than the forces observed in any position of passive limb positioning. In addition, we recognized LHBT internal impingement in the position of the active compression test (forward flexion and internal rotation) in two of six specimens, supporting the hypothesized compressive mechanism of this clinical test. Ultimately, our biomechanical findings provide a thorough data set that may be used to form and/or interpret hypotheses regarding long head biceps tendon function, pathology, and clinical tests.
